# Women With Polycystic Ovary Syndrome: A Review of Susceptibility to Type 2 Diabetes

**DOI:** 10.7759/cureus.33390

**Published:** 2023-01-05

**Authors:** Sakshi Y Layacha, Dalia A Biswas

**Affiliations:** 1 Department of Physiology, Jawaharlal Nehru Medical College, Datta Meghe Institute of Medical Sciences, Wardha, IND

**Keywords:** type 2 diabetes, hyperandrogenism, hyperinsulinemia, polycystic ovary syndrome, insulin resistant

## Abstract

The polycystic ovarian syndrome affects many women today. Previous research has demonstrated a direct link between it and serious ailments such as type 2 diabetes, heart disease, and infertility. Originally thought to be a reproductive disorder, polycystic ovarian syndrome (PCOS) is now understood to be a metabolic and psychological disorder. Women of reproductive age suffering from PCOS undergo hormonal imbalances in which progesterone, insulin, and testosterone are produced in excess. PCOS exhibits a variety of characteristics as well as a heterogeneity of symptoms, including acne, hirsutism, androgenic alopecia, irregular menstruation, infertility, obesity, and mood disorders like despair and anxiety. Chronic anovulation, hyperandrogenism, type 2 diabetes, dyslipidemia, and an elevated threat of coronary artery disease are some of its defining characteristics. PCOS develops due to interacting genetic and environmental factors. From a gynaecological curiosity, it grew into a multisystem endocrinopathy. It is fascinating to learn how hormonal issues result in gynaecological problems. Insulin resistance, compensatory hyperinsulinism, and an increase in ovarian androgenic hyperresponsiveness to circulating insulin are all directly related to hyperandrogenism and anovulation. Independent of weight, insulin resistance is more common with PCOS and plays a crucial role in the syndrome's metabolic and reproductive complications. Anovulation, polycystic ovaries, and elevated luteinizing hormones, which increase circulating androgen, are all caused by a reduction in follicle-stimulating hormone. High androgen levels cause hyperinsulinemia, which leads cells to become insulin resistant and makes PCOS patients more likely to develop diabetes mellitus. Later research established that women with polycystic ovarian shape and persistent anovulation are the only ones susceptible to insulin resistance. Insulin resistance is thus a distinct characteristic of the condition. The purpose of this review paper is to investigate how PCOS ultimately results in type 2 diabetes mellitus.

## Introduction and background

The effects of polycystic ovary syndrome (PCOS) on the body and its correlation with type 2 diabetes mellitus are described here. A prevalent endocrine condition that influences women of fertile age is polycystic ovary syndrome, type 2 diabetes, and hyperlipidemia [1]. PCOS causes an overproduction of the hormones androgen, insulin, and progesterone [2]. Menstrual irregularities, hair loss, acne, and obesity are common symptoms. Understanding how hormonal issues result in gynaecological complaints is quite fascinating. PCOS causes alterations in follicular endocrine signalling; insulin resistance (IR), impaired insulin, and ovarian hyperandrogenism can impair follicular activation, survival, growth, and selection. Small follicles build up around the ovary because of these effects, polycystic morphology emerges, and follicular maturation and anovulation are harmed. Most PCOS sufferers are overweight, obese, or have abdominal obesity [3]. Anovulatory failure, hyperandrogenism, IR, and inflammation are a few PCOS-related dysfunctions that obesity might make worse. As a result, adipogenesis grows and lipolysis declines as these dysfunctions worsen. Changes in the interactions between fat and the ovary, especially when fat is abundant, exacerbate these processes, which harm follicular growth and may impair oocytes [4]. Through the release of numerous inflammatory adipokines and sensitization of thecal cells to IR, obesity can exacerbate IR and inflammation. Therefore, compared to non-obese females with PCOS, obese females with PCOS have many severe phenotypic traits, including oligomenorrhea, sterility, abortion, glucose intolerance, and syndrome X [5].

Patients with hyperinsulinemic PCOS may develop early follicular luteinization because insulin might increase the reactivity of granulosa cells to luteinizing hormone (LH) [6]. Granulosa cells are grown from tiny PCOS follicles that show premature reactivity to LH due to the early development of luteinizing hormone (LH) receptors, which results in increased progesterone synthesis [7]. Because human oocytes include insulin receptors, a premature LH surge aided by excess insulin may also impact oocyte development. Gonadotropin treatment helps patients with hyperinsulinemic PCOS develop more follicles. Relevantly, the cumulus cells of obese PCOS patients express more mRNA for the insulin receptor and specific fatty acid binding proteins than lean PCOS patients [8]. Furthermore, compared to oocytes without PCOS, the insulin receptor (INSR) in oocytes with PCOS may be greater [4].

Anti-Mullerian hormone (AMH) is found in low concentrations in primordial and primary follicles. It reaches its greatest concentration in the large preantral and small antral stages and subsequently decreases in granulosa cells but not cumulus cells throughout the last stages of follicular maturation. The anti-Mullerian hormone is typically produced by these granulosa cells [9]. In ordinary women undergoing in vitro fertilisation (IVF), serum AMH levels positively correlate with the number of antral follicles, serum androgen concentration, and the oocytes retrieved as a marker of developing follicles and negatively connect with the quantity of recombinant human follicle-stimulating hormone (FSH) administered. The follicles of healthy women undergoing IVF similarly do not exhibit a positive relationship between intrafollicular AMH levels and FSH levels [10]. In PCOS, blood AMH and E2 (estradiol) levels are inversely correlated, which is consistent with AMH's ability to reduce FSH receptor mRNA expression and impede FSH-induced aromatase activity in vitro [11]. According to other data, the mRNA levels for AMH, FSH receptor, and androgen are all higher in small and large follicles taken from hormone-stimulated PCOS patients than in control patients. These findings suggest that the inhibitory effects of AMH are diminished or altered in the antral follicles of PCOS patients [12]. IR is a significant contributor to PCOS's excessive adipogenesis and a crucial component of its pathogenesis. In general, IR with severe metabolic abnormalities and late-stage consequences is present in 75% of lean females with PCOS and 95% of obese females with the condition [13]. IR and compensatory hyperinsulinemia affect the pituitary gland, ovaries, and liver and can result in hyperandrogenemia. The release of androgen is substantially higher in the theca cells of PCOS-afflicted women, and insulin has been found to accelerate this process in an in vitro study. In response to adrenocorticotropic hormone stimulation, insulin decreases blood levels of sex hormone binding protein (SHBG), raises free androgen concentration, and raises adrenal androgen production [14].

Recent research revealed that IR and hyperinsulinemia impact most women with PCOS [15]. Given that women with PCOS typically have both ß-cell dysfunction and insulin resistance (IR), the two prerequisites for developing non-insulin-dependent diabetes mellitus, the onset of type 2 diabetes (T2D) in PCOS can be somewhat anticipated. Most women with PCOS have been discovered to have high levels of IR, a crucial participant in the pathophysiology of the disorder, in comparison to their BMI-matched healthy contemporaries. Insulin resistance is a condition in which the activity of the hormone is insufficient to satisfy the metabolic requirements of peripheral tissue, despite an increase in insulin levels. Any insulin-resistant state could conceivably have circulating insulin inhibitors, aberrant and three-cell secretory products, or tissues causing IR [16].

**Figure 1:**
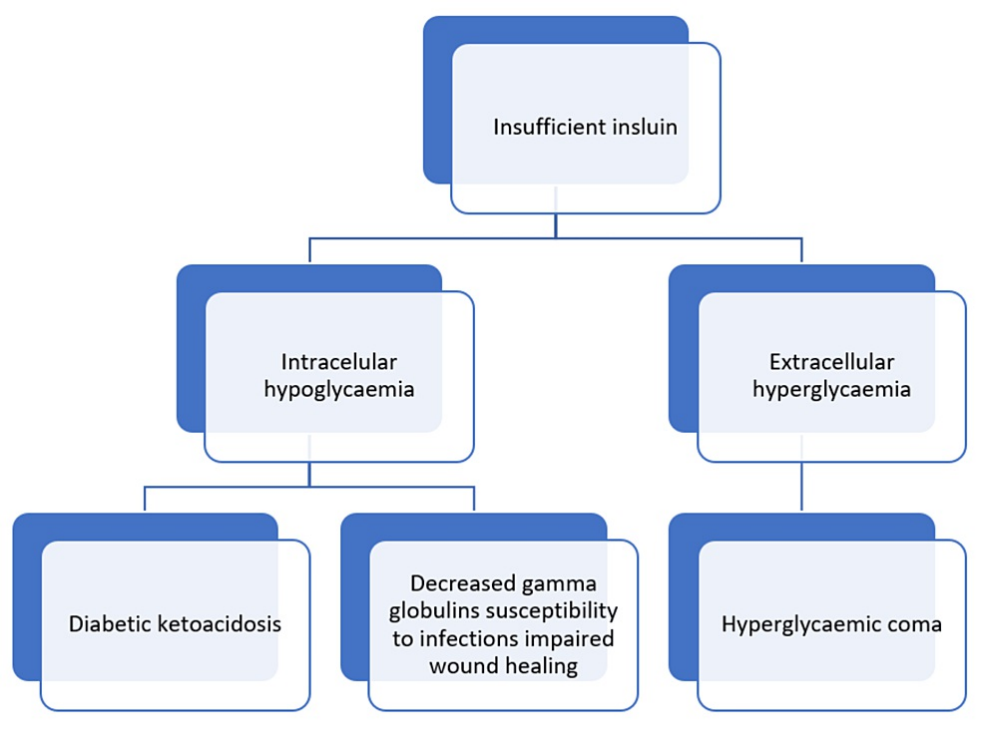
Consequences of insufficient insulin Self-created image

Identifying insulin receptor substrate (IRS) proteins and their role in linking cell surface receptors to intracellular signalling cascades is crucial to understanding the action of insulin and insulin-like growth factor (IGF). Additionally, IRS-proteins integrate signals from the insulin and IGF receptor tyrosine kinases with those produced by proinflammatory cytokines and nutrients. The IRS2 branch of the insulin and IGF signalling cascade has a substantial impact on both the peripheral insulin response as well as the proliferation and function of pancreatic beta-cells. Dysregulation of IRS2 signalling causes peripheral IR in mice, which results in the failure of compensatory hyperinsulinemia. Serine phosphorylation, proteasome-mediated degradation, or chronic stress associated with ageing all inhibit IRS protein signalling and may be essential mediators of IR following acute damage and infection [17]. Figure 1 depicts how insufficient insulin causes diabetic ketoacidosis, decreased gamma globulin susceptibility, and hyperglycaemic coma.

## Review

Methodology

**Figure 2:**
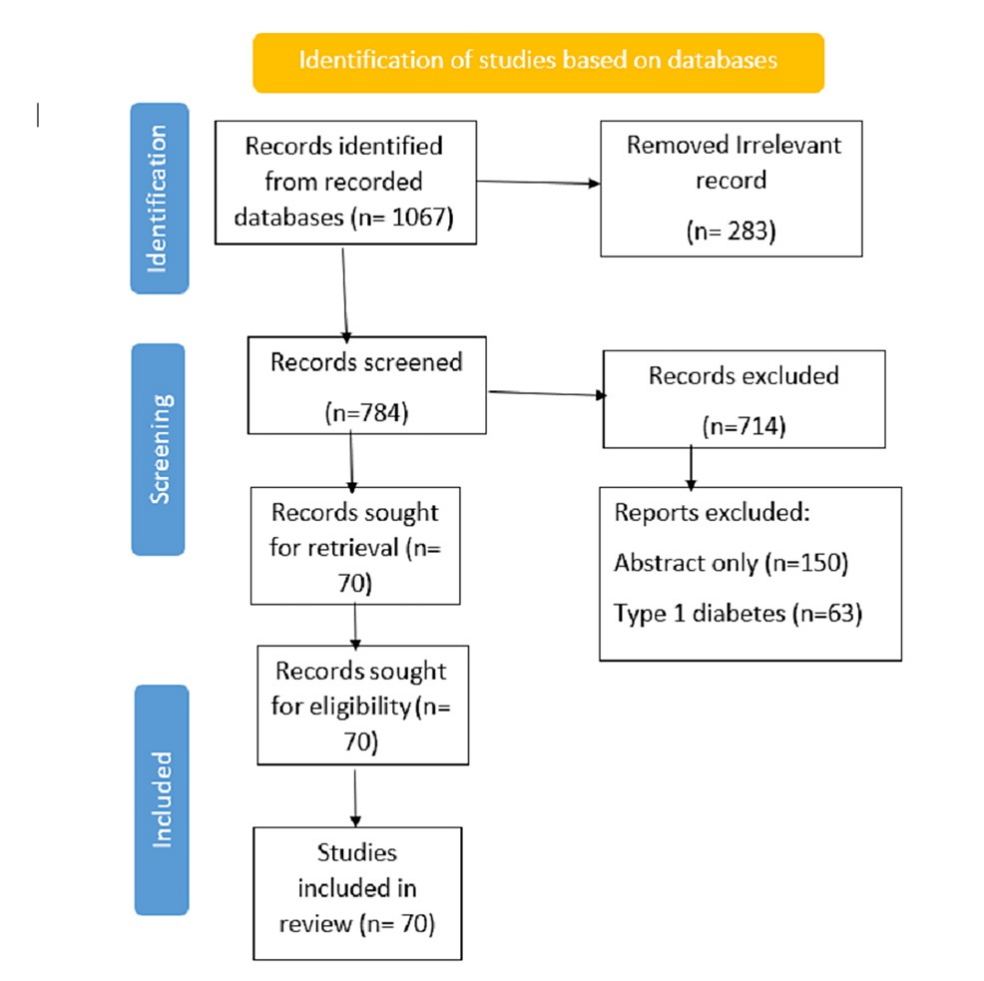
Flowchart on the selection of studies based on PRISMA guidelines PRISMA: Preferred Reporting Items for Systematic Reviews and Meta-Analyses

We undertook a systematic search through PubMed and PubMed Central in August 2022, using keywords such as "polycystic ovary syndrome", "type 2 diabetes", (polycystic ovary syndrome (title/abstract)) OR (PCOS title/abstract) OR (insulin resistant *(title/abstract)) OR ("polycystic ovary syndrome" (MeSH Terms)) AND (("hyperandrogenism" (title/abstract)) OR (type 2 diabetes (title/abstract)) OR (insulin resistant" (MeSH Terms)). We additionally searched for key references in the bibliographies of the relevant studies. The search was updated in November 2022. Reviewers independently monitored the retrieved studies against the inclusion criteria, first based on the title and abstract and then on full texts. The flowchart reports the number of records identified from each database or registered search; it also indicates how many records were excluded, as shown in Figure 2.

Polycystic ovary syndrome

Polycystic ovary syndrome (PCOS) causes a hormonal imbalance with an increased amount of androgen, luteinizing hormone, and insulin. It shows various symptoms like acne, hirsutism, irregular menstruation, and obesity. Besides these, it is accompanied by hyperinsulinemia, hyperandrogenism, hyperlipidemia, anovulation, and insulin resistance [18]. An excess amount of androgen, which has ovarian and adrenal origins, leads to the symptoms of seborrhoea, alopecia, acne, and hirsutism; the most successful way to treat this condition is with anti-androgens. Premature births, neonatal problems, foetal malformations, miscarriages, and issues throughout pregnancy are all made more likely by it.

Additionally, PCOS encourages psychological morbidity, including sadness, a negative view of one's body and self, and a decline in a healthy lifestyle. Therefore, PCOS is linked to both short- and long-period manifestations that may negatively impact females at different times of their lives. To lessen the syndrome's financial and health costs, it is crucial to find effective medicines for its prevention and treatment [19]. Hyperandrogenism and anovulation are related to insulin sensitivity, compensatory hyperinsulinemia, and a rise in the ovarian androgenic response to circulating insulin. Although metabolic disorders and PCOS have traditionally been thought to be related, there is increasing knowledge of the crucial role that insulin resistance plays in the endocrine and reproductive problems associated with the disease. Several hypotheses have been tested to clarify how PCOS is associated with an increased threat of developing insulin resistance. This may be somewhat described by the fact that PCOS is frequently linked to obesity, typically accompanied by insulin resistance. However, many women with PCOS are insulin-resistant even when not obese [20]. This is because these women are at increased risk of developing diabetes even if they are not obese [21]. All obese women with PCOS are still asked to make lifestyle changes as their first line of treatment. However, many of these women struggle to lose weight.

Insulin resistance

Insulin resistance occurs when the increased amount of insulin produced and put into circulation cannot meet the metabolic needs of peripheral tissue. According to research, the first women with PCOS were diagnosed with hyperinsulinemia around 1980 [22]. Subsequently, it was noted that many women with PCOS had mild acanthosis, a skin indicator of insulin resistance. Only females with persistent anovulation and polycystic ovary morphology exhibit insulin resistance, according to recent studies. Thus, insulin resistance serves as a defining characteristic of the disease known as persistent anovulation. Metformin treats insulin resistance; it can increase fertility, help people lose weight, boost their lipid profiles, and decrease their risk of developing diabetes, myocardial infarction, and stroke.

Numerous phenotypic and clinical characteristics of women with polycystic ovary syndrome (PCOS) may serve as a reference for therapeutic alternatives for metabolic protection and ovulation induction. Metformin use may be beneficial for some PCOS-affected female patients. Women with PCOS should take precautions for the development of type 2 diabetes mellitus since this condition is indicated by raised insulin levels on a two-hour 75-g glucose tolerance test (DM). Metformin medication may potentially reduce cardiovascular risk factors, such as dyslipidemia and signs of subclinical inflammation. For ovulation induction in PCOS women, metformin is less effective than clomiphene citrate [23]. In PCOS-afflicted women, a 6-week prescription of metformin may enhance menstrual cyclicity and fertility. A logical strategy for treating the biochemical and hormonal problems in PCOS women is provided by insulin-sensitizing medications [24].

Insulin resistance is a distinctive feature of polycystic ovary syndrome. It is caused by a post-binding error in signalling that persists in cultured skin fibroblasts and is linked with constitutive serine phosphorylation on the insulin receptor. One reason for the post-binding impairment in insulin action diagnostic of PCOS is increased serine phosphorylation, which lowers the activity of tyrosine kinase. Therefore, it is interesting that insulin receptor autophosphorylation is a crucial factor in this resistance, based on several studies [25]. After investigating numerous PCOS patients, it was found that PCOS-related cells had lower insulin resistance and tyrosine phosphorylation. Fibroblasts from PCOS patients were pre-treated with a serine kinase inhibitor to investigate the function of elevated serine kinase activity in a decline of I.R. autophosphorylation in PCOS. Serine phosphorylation of insulin receptor substrate (IRS)-1 and other downstream signalling components is possible. In cultured myotubes from PCOS-affected women, basal and insulin-stimulated glucose transport, as well as glucose transporter 1 (GLUT1) abundance, were all considerably elevated. The levels of tyrosine autophosphorylation, insulin receptor substrate (IRS)-1-associated IR-subunits, and phosphatidylinositol (PI) 3-kinase activity were comparable between the two groups. On the other hand, IRS-1 protein levels in excess dramatically rose in PCOS when PI 3-kinase activity was corrected. People with PCOS have skeletal muscles with an acquired deficit that reduces the amount of glucose absorbed when insulin is activated. Myotubes from affected women, however, show innate flaws in insulin signalling and glucose transport, including elevated phosphorylation of IRS-1 Ser312, which may increase vulnerability to factors that promote insulin resistance. These aberrations are distinct from those reported in other insulin-resistant states. This is consistent with the hypothesis that PCOS is a genetically separate syndrome that raises the risk for type 2 diabetes. [26].

Studies on hyperandrogenic teenage and adult PCOS women have found a clear link between insulin resistance, or hyperinsulinemia, and anovulation and biochemical hyperandrogenaemia. A proper investigation revealed that a patient's testosterone level was reduced concurrently with increased insulin sensitivity. Androgen may directly affect the signalling cascade, causing insulin action to be impaired. The signalling flaw specifically affected the insulin metabolic pathway and resulted in reduced protein kinase phosphorylation [27].

Long-term PCOS treatment requires modifying additional lifestyle factors, including smoking, alcohol use, and psychosocial stressors [28]. Lifestyle changes are the mainstay of PCOS treatment; however, they are not very durable or adherent. Some, but not all, anovulatory people have shown improved ovarian responsiveness to gonadotropins after receiving growth hormone treatment. Treatment with growth hormones has been shown to partially regulate follicular growth, which is typically halted in PCOS. In addition to the injection of growth hormone, therapeutic methods to modify the growth hormone-ovarian axis are being studied. High serum luteinizing hormone levels are frequently linked to PCOS. These are commonly connected to infertility and early pregnancy loss. Contrary to what is achieved with clomiphene citrate, the result of pregnancies is improved by lowering the levels of luteinizing hormone in conjunction with gonadotropins. The prior treatment plan may cause ovarian hyperstimulation in PCOS individuals [29]. Steatosis is more common in obese women with PCOS. Anti-androgen therapy is used in conjunction with medications that increase insulin sensitivity to treat PCOS medically. While combined oral contraceptives are frequently used as anti-androgens, spironolactone may also have vascular advantages. Glucagon-like peptide-1 (GLP-1) agonists, Dipeptidyl Peptidase-4 (DPP-4) inhibitors, Sodium-Glucose Transport Protein-2 (SGLT2) inhibitors, myoinositol, thyroid hormones, and vitamin supplements are among the new and forthcoming treatments for PCOS. Although statin drugs are rarely recommended for treating high cholesterol in PCOS, they may ameliorate the phenotype of the condition. Antidepressant use in PCOS patients may negatively impact their metabolic phenotype [30].

More noteworthy than the percentage of PCOS patients who have insulin resistance is that these women have a higher risk of developing metabolic syndrome (syndrome X). The traits of this disorder include dyslipidemia, abdominal obesity, coagulopathy, poor fibrinolysis, a greater risk of developing high blood pressure, type II diabetes, and heart disease. Hyperglycemia has been linked to a profile of heightened cardiovascular risk factors in PCOS, according to research [31]. It was found that older women with PCOS had a higher burden of atherosclerosis than normal women by comparing the carotid intimal medial thickness on ultrasound to that of normal women. The typical nocturnal decline in blood pressure does not occur in adolescents with PCOS and impaired fasting glucose.

Insulin resistance (IR) is an insulin resistance syndrome in which the peptide hormone insulin has a physiologic effect on peripheral target tissues that is less than predicted, resulting in hyperinsulinemia, the characteristic feature of IR [32]. IR is frequently linked to the consequences of obesity, including type 2 diabetes, PCOS, cardiovascular disease, some forms of cancer, and infertility [33]. Energy-reduced meals, when incorporated into a healthier diet, may nearly always aid in losing weight and minimising IR in these patients, even when surgical and pharmaceutical methods have been demonstrated to be effective [34]. In obese people, even a modest weight loss of 5%-10% can result in several good health outcomes, such as an improvement in cardiometabolic parameters, a decrease in blood pressure, and an extension of life [35].

The connection between hyperinsulinemia and insulin resistance in PCOS has to be further investigated. Researchers are currently looking for information to understand the origin of this mysterious illness.

## Conclusions

We infer that type 2 diabetes is more likely in women with PCOS. It is brought on by hormonal imbalance, which interferes with the insulin receptor and follicle formation. Even though the body produces a lot of insulin, cells resist it, making them more vulnerable to diabetes. Recent research has proven that women with polycystic ovary syndrome and persistent anovulation are the only ones who are insulin resistant. Thus, chronic anovulation syndrome has insulin resistance as a distinctive hallmark. Drugs such as insulin sensitizers are discussed as an optimistic and peculiar curative option for the chronic treatment of PCOS. It is important to note that polycystic ovaries are not a requirement for PCOS, and many women with them do not have the condition. If incidental polycystic ovaries are discovered on an ultrasound for another reason, PCOS should not be assumed to be present unless there is corroborating clinical evidence.
